# Method development for cross-study microbiome data mining: Challenges and opportunities

**DOI:** 10.1016/j.csbj.2020.07.020

**Published:** 2020-08-01

**Authors:** Xiaoquan Su, Gongchao Jing, Yufeng Zhang, Shunyao Wu

**Affiliations:** a College of Computer Science and Technology, Qingdao University, Qingdao, Shandong 266071 China; b Single-Cell Center, Qingdao Institute of BioEnergy and Bioprocess Technology, Chinese Academy of Sciences, Qingdao, Shandong 266101 China

**Keywords:** Microbiome, Shotgun metagenome, Amplicon sequencing, Data mining, Microbiome search, Multi-omics data

## Abstract

During the past decade, tremendous amount of microbiome sequencing data has been generated to study on the dynamic associations between microbial profiles and environments. How to precisely and efficiently decipher large-scale of microbiome data and furtherly take advantages from it has become one of the most essential bottlenecks for microbiome research at present. In this mini-review, we focus on the three key steps of analyzing cross-study microbiome datasets, including microbiome profiling, data integrating and data mining. By introducing the current bioinformatics approaches and discussing their limitations, we prospect the opportunities in development of computational methods for the three steps, and propose the promising solutions to multi-omics data analysis for comprehensive understanding and rapid investigation of microbiome from different angles, which could potentially promote the data-driven research by providing a broader view of the “microbiome data space”.

## 1 Introduction

Microbiome data provides a unique view to understand the micro-ecology and further investigate the interactions between microorganisms and their surrounding environment [1]. In recent years, a vast number of microbial community specimens have been sequenced to study on the microbial- associations to the natural environment dynamics [2], [3], human health [4], [5], [6], [7], agriculture [8], [9], etc. Therefore, how to efficiently and comprehensively discover biological stories hidden under such a large-scale data has become one of the most essential bottlenecks for microbiome research at present [10], [11]. Newly developed bioinformatics tools are bringing opportunities in deciphering the microbiome data, from general-purpose algorithms such as sequence alignment and machine learning (ML), to microbiome-specific approaches like operational taxonomy unit (OTU) picking [12] and phylogeny-based distance metrics [13], [14]. On the other hand, challenges have also already been placed by the vast volume of microbiome data, especially in integration of datasets produced by multiple studies and platforms [15], comparison among samples [16] and status or disease classification and prediction by training on large-scale datasets [17], [18].

**Fig. 1:**
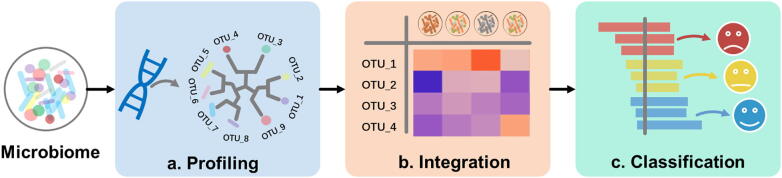
**Key steps for *meta*-analysis on cross-study microbiome big-data.** (**a**) Compositional profiling decodes the microbiome taxonomical and functional profiles from sequences. (**b**) Data integration curates, normalizes and unifies existing datasets. (**c**) Data mining identifies and classifies the status of a given specimen by learned microbial features from integrated data.

Meta-analysis on cross-study datasets can generate constant and reproducible results as fundamental for further studies and applications [19], [20], [21]. Three analytical steps (Fig. 1) are playing crucial roles in handling microbiome big-data: *compositional profiling* that decodes the microbiome taxonomical and functional profiles from sequences (Fig. 1**a**), *data integration* that curates, normalizes and unifies existing datasets (Fig. 1**b**), and *data mining* that identifies and classifies the status of a given specimen by learned microbial features from integrated data (Fig. 1**c**). By reviewing the computational methods and tools development for microbiome profiling, integration and data mining respectively, in this mini-review we summarize the challenges and opportunities from such three aspects (Table 1 and Table 2), and propose more prospective solutions for comprehensive understanding and rapid investigation of microbiome from different angles by multi-omics data analysis.

**Table 1:** Summary of challenges and opportunities for microbiome data analysis.

Methods	Major challenges and limitations	Opportunities and prospects
Microbial profiling	**Marker-based profiling** • Only genus-level resolution • Limited applicable range for functional profiling **WGS-based profiling** • Expensive sequencing cost • Both data- and computing-intensive for analysis	**Full-length 16S** • Species- or strain-level resolution • Expanded marker-genome linkage • Unified reference and definite phylogeny for wide-range comparison **Shallow WGS** • Obtain species-level taxonomic and functional data at approximately the same cost as amplicon sequencing
Data integration	**General-purpose repositories** • Mostly only store raw sequences • Lack of unified metadata and annotation • Difficult to seek microbiomes that under a targeted condition or with specific features	**Curated database** • Standard sequence quality control • Unified microbial structural profiles and metadata annotation • Feature-based sample query **Microbiome search engine** • “Community to communities” match on whole-microbiome-level • Real-time level search speed
Status classification and prediction	**Machine learning** • Difficult to broadly decide whether a microbiome is healthy or not • Inadequate performance in multiple-status classification • Hard to extend a model to other cohorts	**Search-based approach** • Status-assumption-free and bio-marker-free • Robustness to data heterogeneity and contamination **Deep learning** • Hardware and system environment support for big-data training • Optimization in multi-tag classification • Well-implemented script-based packages

**Table 2:** Summary of current tools for microbiome data analysis.

Tool name	Type	URL	Parallel computing	Installation	Reference
UParse	OTU clustering tool	https://drive5.com/uparse/	Multi-threads parallel computing	Binary package	[12]
Usearch	Integrated sequence analysis tool for amplicons (e.g. OTU clustering, denoising)	https://www.drive5.com/usearch/	Multi-threads parallel computing	Binary package	[23]
Vsearch	Alternative implementation of Usearch	https://github.com/torognes/vsearch	Multi-threads parallel computing	Source code / Binary package	[49]
DADA2	Amplicon sequence variants (ASVs) tools	https://benjjneb.github.io/dada2/	Multi-threads parallel computing	Bioconda / Source code / Binary package	[24]
Deblur	Amplicon sequence variants (ASVs) tools	https://github.com/biocore/deblur	Multi-threads parallel computing	Conda / Source code	[25]
UNOISE3	Amplicon sequence variants (ASVs) tools	http://www.drive5.com/usearch/manual/unoise_algo.html	Multi-threads parallel computing	Binary package	[26]
PICRUSt/PICRUSt2	Functional profiles prediction from amplified marker genes	http://picrust.github.io/picrust/	Multi-threads parallel computing	Bioconda / Miniconda / Source code / Online service (galaxy)	[28], [29]
Tax4Fun	Functional profiles prediction from amplified marker genes	http://tax4fun.gobics.de/	Not appliable	R package	[30]
QIIME/QIIME2	Integrated microbiome bioinformatics workflow	http://qiime.org/https://qiime2.org/	Partially with multi-thread parallel computing, depends on the specific tool in the pipeline	Conda / Miniconda / VirtualBox / Docker	[31], [32]
Mothur	Integrated microbiome bioinformatics workflow	https://mothur.org/	Partially with multi-thread parallel computing, depends on the specific tool in the pipeline	Binary package / Source code	[33]
Parallel-META3	Integrated microbiome bioinformatics workflow	http://bioinfo.single-cell.cn/parallel-meta.html	Multi-threads parallel computing	Source code	[34]
Karken	Taxonomical annotation of WGS short reads	http://ccb.jhu.edu/software/kraken/	Multi-threads parallel computing	Source code	[40]
mOTUs	Taxonomical annotation of WGS short reads	https://motu-tool.org/	Multi-threads parallel computing	Conda / Source code	[41]
MetaphlAn2	Taxonomical annotation of WGS short reads	https://huttenhower.sph.harvard.edu/metaphlan	Multi-threads parallel computing	Bioconda / Source code	[42]
HUMANn2	Functional annotation of WGS short reads	https://huttenhower.sph.harvard.edu/humann	Multi-threads parallel computing	Source code / Python-pip / Conda	[43]
metaSPAdes	Assembling of WGS short reads	https://github.com/ablab/spades	Multi-threads parallel computing	Source code / Binary package	[44]
Meta-IDBA	Assembling of WGS short reads	https://github.com/loneknightpy/idba	Multi-threads parallel computing	Source code	[45]
MetaWARP	Extraction and interpretation of high-quality metagenomic bins	https://github.com/bxlab/metaWRAP	Partially with multi-thread parallel computing, depends on the specific tool in the pipeline	Conda / Bioconda / Docker / Source code	[46]
NCBI-SRA	Online general-purpose bio-data repository	https://www.ncbi.nlm.nih.gov/sra	Not appliable	Online service	[57]
MG-RAST	Online microbiome data repository	https://www.mg-rast.org/	Not appliable	Online service	[58]
EBI-Metagenomics	Online microbiome data repository	https://www.ebi.ac.uk/metagenomics/	Not appliable	Online service	[59]
JGI-IMG/M	Online microbiome data repository	https://img.jgi.doe.gov/	Not appliable	Online service	[60]
MPD	Pathogen genome and metagenome database	http://data.mypathogen.org	Not appliable	Online service	[61]
GMrepo	Curated database of human gut metagenomes	https://gmrepo.humangut.info/home	Not appliable	Online service	[65]
GcMeta	Integrated microbiome research platform	https://gcmeta.wdcm.org/	Partially with multi-thread parallel computing, depends on the specific tool in the pipeline	Online service	[66]
Qiita	Online microbiome study management platform	https://qiita.ucsd.edu/	Partially with multi-thread parallel computing, depends on the specific tool in the pipeline	Online service	[67], [68]
MSE	Microbiome search engine	http://mse.ac.cn/	Multi-threads parallel computing	Online Service / Source code	[69]
TensorFlow	Open source platform for machine learning	https://www.tensorflow.org/	GPU parallel computing	Python-Pip / Docker / Source code	
PyTorch	Library for deep learning	https://pytorch.org/	GPU parallel computing	Conda / Python-pip / Source code	
IMP	Integrated *meta*-omic pipeline framework	https://r3lab.uni.lu/web/imp/	Partially with multi-thread parallel computing, depends on the specific tool in the pipeline	Conda / Docker / Source code	[97]

## 2 Microbiome compositional profiling

DNA sequencing is the primary approach to survey the compositional features of microbial communities [22]. Generally, two sequencing strategies are widely used: amplicon sequencing that employs the marker genes (e.g. 16S rRNA, 18S rRNA or ITS) for taxonomy identification, and shotgun metagenomic whole-genome sequencing (WGS) that captures genome-wide sequences of all organisms in a sample.

For marker-gene-based analysis, several algorithms have been widely used for taxonomy assignment by sequence clustering and OTU picking algorithms like UPARSE [12] and Usearch [23] that based on sequence similarity. Amplicon sequence variants (ASVs) tools such as DADA2 [24], Deblur [25] and UNOISE3 [26] are further developed to improve the analytical precision of amplicon sequences on single-nucleotide level, which have higher reliability, reproducibility and comprehensiveness than regular OTUs [27]. Functional profiles could also be inferred from amplicons using the linkages between marker genes and reference genomes by PICRUSt [28], [29], Tax4Fun [30] and other similar software. Most of these approaches have already been integrated into comprehensive pipelines such as QIIME [31], [32], Mothur [33] or Parallel-META3 [34] with additional statistical processes for quantitative analysis on alpha and beta diversity of microbial communities. As a cost-efficient method, amplicon-based analysis has been adopted for large-scale microbiome surveys, however, the accuracy is also limited due to PCR bias [35], low-resolution of short-read-based markers and lack of marker-genome associations. For example, taxonomy annotation by targeting sub-regions of 16S rRNA short-reads is always on genus level [36], [37], and function prediction is not accurate for environmental microbes that lack reference genomes [28].

Since WGS is more informative, some approaches utilize unassembled WGS short reads for species or strain level taxonomy annotation [38], [39] (e.g. Karken [40], mOTUs [41], and MetaPhlAn2 [42]) and direct function parsing (e.g. HUMANn2 [43]), as well as binning- or assembling-based tools (e.g. metaSPAdes [44], *meta*-IDBA [45] and MetaWRAP [46]) are capable for species genome re-construction, *de novo* gene prediction and single nucleotide polymorphism (SNP) analysis. Nevertheless, WGS is also limited for a broad-range application by the 3–10 folds higher overall cost including sequencing, data storage and sharing, bioinformatics processing of reads quality control [47], [48], taxonomical and functional [38], [43] profiling than those of amplicons [28], [34], [49], [50]. A new library preparation protocol of shallow shotgun sequencing obtains species-level taxonomic and functional profiles of microbiomes similar to that offered by regular deep sequencing, making the WGS in a more economical way [51].

Rather than targeting specific variable sub-regions of short-read-based amplification, full-length 16S rRNA gene sequencing by PacBio or Oxford Nanopore sequencing platforms has the potential for accurate classification of individual organisms from microbial communities at species or strain taxonomic resolution [52]. Meanwhile, since more and more full-length 16S rRNA gene sequences and full genomes have been released [53], mapping markers to unified references also enables the high-resolution comparison of microbiome profiles on a wide range. To couple with such advantages by long-read sequencing platform data, new denoising, sequence clustering and annotation algorithms and strategies should also be updated. Thus, the rapid development of microbiome profiling methods provides the basis to enable a broader view of the “microbiome data universe”.

## 3 Data repositories and integration

A huge number of microbiome datasets have been produced by studies such as Human Microbiome Project [54], Earth Microbiome Project [55] and American Gut Project [56]. Samples have been deposited in online repositories, e.g. NCBI-SRA [57], MG-RAST [58], EBI Metagenomics [59], JGI-IMG/M [60], MPD [61] and so on. Such massive data brings the “materials” for research on the global-wide microbial diversity and distribution, while also makes new problems in data integration and reusage. In these repositories, most samples are organized by study and stored as raw or clean DNA sequences, and metadata among studies are not unified for feature selection and comparison, leading to the difficulty for seeking microbiomes under a targeted condition or with specific features.

To utilize and reuse valuable microbiome big-data for further *meta*-analysis and comparison, several works re-organized the microbiome samples with unified metadata format [62], [63] and standard operating procedures (SOPs) [64] for sequence processing. GMrepo [65] is a database of well-organized and curated human gut metagenomes with constantly annotated metadata. GcMeta [66] features a data management system that integrated with data analysis tools and workflows for archiving and publishing data in a standardized way. In addition, Qiita [67], [68] allows users to perform *meta*-analysis across multiple studies, and retrieve microbiomes that contain a specific feature (e.g. metadata, taxon terms, and sequence fragments) by SQL-like queries.

Nevertheless, when new microbiomes are sequenced, it is still difficult to find what existing microbiomes in the repositories or databases have overall similar composition to them, thus answer further questions like prediction of environmental conditions or human health status. To tackle this case, a Microbiome Search Engine (MSE) [69] has been developed for rapid “community to communities” comparisons and matches. By a dynamic indexing strategy and a series of whole-microbiome-level similarity scoring function [70], [71], MSE enables the real-time-level accessibility of targeted microbiomes with specific structure from massive volume of data.

Another important barrier for integrating the cross-study microbiome datasets is the technical variation of amplicon sequencing data from multiple sources and batches. Technical factors can significantly affect the comparison among datasets including DNA extraction, PCR primers for marker genes, sub-regions of the marker gene amplification, sequencing platforms and types of sequence reads [72]. For biological studies with large effect size like comparing environmental microbiomes from multiple habitat types, human microbiomes from different body sites and from hosts with different ages, locations and diets, the technical differences can be outweighed by referenced-based taxonomy assignment of 16S rRNA (e.g. mapping short-reads to full-length 16S rRNA genes) [73], [74], making the cross-study integration to be meaningful. However, studies of more subtle effects still require unified experimental protocols for producing amplicon datasets. In contrast, shotgun WGS has been tested as less sensitive to technical differences in studying the disease association and temporal dynamics of microbiome [19], [75], which is an alternative option for integration and comparison of cross-study datasets.

## 4 Data mining for status identification and classification

Since microbial communities shape the dynamics of ecological systems, ranging from the human gut to the marine, one potential of microbiome is linking variation of microbial composition to phenotypic and physiological statuses, which can inspire the development of new techniques for disease diagnosis, ecological dysbiosis detection and treatment evaluation. Previous studies have demonstrated the feasibility of ML methods [18], [76] in disease detection and classification with human-associated microbiome data for inflammatory bowel disease (IBD) [77], colorectal cancer (CRC) [19], caries [78], etc., by extreme gradient boosting (XGBoost), random forest (RF), support vector machine (SVM), k-nearest neighbor (KNN) and other ML algorithms. As a quantitative approach, the ML-based indices are also designed to assess the risks for potential diseases and to evaluate the effects among different treatments [79], [80].

Typically, microbiome-based detection has to make *a priori* assumption about a specific status (e.g. a disease) for given samples, and seek organismal or functional features (e.g. taxon or gene) that unevenly distributed between disease and control samples as bio-markers. Then ML models are trained and constructed using these bio-markers for disease recognition. Since the detection range is restricted to the given status types in such models, it is difficult to broadly decide whether the sample is healthy or not. Furthermore, extending a particular model of a disease to other cohorts can be challenging due to the heterogeneity of microbiome data among population [81]. In addition, the same bio-markers can be associated with multiple different diseases, which may also result in errors in multiple disease classification [82].

A search-based strategy for disease detection and classification has been developed, which detects abnormal samples via their outlier search-based novelty against large number of samples from healthy subjects, and then identifies the specific disease type by top-hits that searched in samples from patients [83]. This whole-microbiome-level search and match strategy enables the identification of microbiome states associated with disease even in the presence of different cohorts, multiple sequencing platforms or significant contamination, while currently the software is only implemented for amplicon sequences processed by referenced OTU picking.

Nowadays, application of deep learning such as deep neural network (DNN) or convolutional neural network (CNN) has been shifted from computer vision problems to microbial biological field [17]. By parallel-computing-based hardware-level boost of multi-core CPU and many-core GPU, deep learning approach shows its advantages in big data integration and robustness to data heterogeneous [84], while the particular parameters in model construction still need to be optimized for solving different questions. At the same time, TensorFlow (https://www.tensorflow.org/) and PyTorch (https://pytorch.org/) packages provide the easy implementation of artificial intelligence (AI) techniques by Python, driving the applications of deep learning for microbial analysis in taxonomy identification [85], biomarker selection [86], multiple disease detection and classification [87]. Another potential of deep learning in microbiome research is the ability of multi-label classification that has been widely used in image processing [88]. It is common that a single microbiome specimen could be associated with more than one disease, and such samples have been collected by several studies [56], [89]. Since the current studies on microbiome and disease mainly focus on single-label classification that each individual sample is only with one specific status, such situation could be solved by further extension of AI techniques in microbiome field.

## 5 Outlook of multi-omics data analysis

Studying on “what organisms exist in a microbial community” and “what a microbial community can do” is no longer adequate to fully understand the interactions between microbiome and environment. Although the profiling of DNA sequencing surveys the functional genes in a microbial community, the functional activities and gene expressions of cells and the metabolite products that reflect the biosynthetic features are still unclear. Multi-omics data analysis of microbiome [90] utilizes chemical and biological approaches to provide a comprehensive view on “what a microbial community is doing”, which investigates a microbiome community from further dimensions of metatranscriptomics [91], metaproteomics [92], metabolomics [93] and viromics [94]. Some of the previous works have demonstrated the in-depth and unique insights of multi-omics data in understanding human microbiome [95], [96]. Nevertheless, the data types and computational tools are mostly omics-specific, e.g. software for metagenomic sequencing is not compatible with RNA-seq data of metatranscriptomics and mass spectrum data of metabolomics, making the combination of the multiple tools to be case-specific, inextensible and irreproducible. Recently, a workflow named IMP (Integrated Meta-omic Pipeline) was released to perform automatic, standardized and flexible analysis to incorporate metagenomic and metatranscriptomic data [97]. This open-development framework strategy enhances the integration of different type data analysis and the interpretation of results from multiple aspects, as well as promotes the general paradigm of microbiome multi-omics research.

Sequencing-based analysis is not routinely used in clinical or industrial applications mainly due to the data generation by sequencers usually takes at least 2 days [98]. At present, fluorescence-activated cell sorting (FACS) approaches have been developed for rapid functional cell-sorting, which is based on the labeling of cells for target proteins, metabolites, or nucleic acids [99]. A new series of label-free, single-cell-level imaging tools using Raman-activated cell sorting (RACS) are also proposed for the taxonomy or status identification of individual cells in a microbial community [100], [101]. Because it is an imaging approach, obtaining the Raman spectrum can be non-destructive to the cell and does not require external labeling or preexisting biomarkers. More importantly, since FACS or RACS only costs seconds to profile each cell, such techniques can be considered as single-cell-resolution approaches that monitor microbiome with high throughput and low time cost.

## Declaration of Competing Interest

The authors declare that they have no known competing financial interests or personal relationships that could have appeared to influence the work reported in this paper.

## References

[1] Blaser M.J. (2016). Toward a Predictive Understanding of Earth's Microbiomes to Address 21st Century Challenges. mBio.

[2] Bork P. (2015). Tara Oceans. Tara Oceans studies plankton at planetary scale Introduction. Science.

[3] Wu L. (2019). Global diversity and biogeography of bacterial communities in wastewater treatment plants. Nat. Microbiol..

[4] Forslund K. (2015). Disentangling type 2 diabetes and metformin treatment signatures in the human gut microbiota. Nature.

[5] Halfvarson J. (2017). Dynamics of the human gut microbiome in inflammatory bowel disease. Nat. Microbiol..

[6] Poore G.D. (2020). Microbiome analyses of blood and tissues suggest cancer diagnostic approach. Nature.

[7] Qin J. (2010). A human gut microbial gene catalogue established by metagenomic sequencing. Nature.

[8] Gao P. (2017). Feed-additive probiotics accelerate yet antibiotics delay intestinal microbiota maturation in broiler chicken. Microbiome.

[9] Zhang J.Y. (2019). NRT1.1B is associated with root microbiota composition and nitrogen use in field-grown rice. Nat. Biotechnol..

[10] Kyrpides N.C., Eloe-Fadrosh E.A., Ivanova N.N. (2016). Microbiome Data Science: Understanding Our Microbial Planet. Trends Microbiol..

[11] Wood-Charlson E.M. (2020). The National Microbiome Data Collaborative: enabling microbiome science. Nat. Rev. Microbiol..

[12] Edgar R.C. (2013). UPARSE: highly accurate OTU sequences from microbial amplicon reads. Nat. Methods.

[13] Lozupone C., Knight R. (2005). UniFrac: a new phylogenetic method for comparing microbial communities. Appl. Environ. Microbiol..

[14] Su X., Xu J., Ning K. (2012). Meta-Storms: Efficient Search for Similar Microbial Communities Based on a Novel Indexing Scheme and Similarity Score for Metagenomic Data. Bioinformatics.

[15] Sinha R. (2017). Assessment of variation in microbial community amplicon sequencing by the Microbiome Quality Control (MBQC) project consortium. Nat. Biotechnol..

[16] Comin M. (2020). Comparison of microbiome samples: methods and computational challenges. Brief Bioinform.

[17] Cammarota G. (2020). Gut microbiome, big data and machine learning to promote precision medicine for cancer. Nat. Rev. Gastroenterol. Hepatol..

[18] Goecks J. (2020). How Machine Learning Will Transform Biomedicine. Cell.

[19] Wirbel J. (2019). Meta-analysis of fecal metagenomes reveals global microbial signatures that are specific for colorectal cancer. Nat. Med..

[20] Bisanz J.E. (2019). Meta-Analysis Reveals Reproducible Gut Microbiome Alterations in Response to a High-Fat Diet. Cell Host Microbe.

[21] Armour C.R. (2019). A Metagenomic Meta-analysis Reveals Functional Signatures of Health and Disease in the Human Gut Microbiome. mSystems.

[22] Knight R. (2018). Best practices for analysing microbiomes. Nat. Rev. Microbiol..

[23] Edgar R.C. (2010). Search and clustering orders of magnitude faster than BLAST. Bioinformatics.

[24] Callahan B.J. (2016). DADA2: High-resolution sample inference from Illumina amplicon data. Nat. Methods.

[25] Amir A. (2017). Deblur Rapidly Resolves Single-Nucleotide Community Sequence Patterns. mSystems.

[26] Edgar R.C. (2016). UNOISE2: improved error-correction for Illumina 16S and ITS amplicon sequencing. bioRxiv.

[27] Callahan B.J., McMurdie P.J., Holmes S.P. (2017). Exact sequence variants should replace operational taxonomic units in marker-gene data analysis. ISME J.

[28] Langille M.G. (2013). Predictive functional profiling of microbial communities using 16S rRNA marker gene sequences. Nat. Biotechnol..

[29] Douglas G.M. (2020). PICRUSt2 for prediction of metagenome functions. Nat. Biotechnol..

[30] Asshauer K.P. (2015). Tax4Fun: predicting functional profiles from metagenomic 16S rRNA data. Bioinformatics.

[31] Caporaso J.G. (2010). QIIME allows analysis of high-throughput community sequencing data. Nat. Methods.

[32] Bolyen, E., et al., Reproducible, interactive, scalable and extensible microbiome data science using QIIME 2 (vol 37, pg 852, 2019). Nat. Biotechnol., 2019. 37(9): p. 1091-1091. 10.1038/s41587-019-0209-9 PMC7015180 31341288

[33] Schloss P.D. (2009). Introducing mothur: open-source, platform-independent, community-supported software for describing and comparing microbial communities. Appl. Environ. Microbiol..

[34] Jing G. (2017). Parallel-META 3: Comprehensive taxonomical and functional analysis platform for efficient comparison of microbial communities. Sci. Rep..

[35] Jones M.B. (2015). Library preparation methodology can influence genomic and functional predictions in human microbiome research. PNAS.

[36] Edgar R.C. (2018). Accuracy of taxonomy prediction for 16S rRNA and fungal ITS sequences. Peer J..

[37] Yarza P. (2014). Uniting the classification of cultured and uncultured bacteria and archaea using 16S rRNA gene sequences. Nat. Rev. Microbiol..

[38] Ye S.H. (2019). Benchmarking Metagenomics Tools for Taxonomic Classification. Cell.

[39] Scholz M. (2016). Strain-level microbial epidemiology and population genomics from shotgun metagenomics. Nat. Methods.

[40] Wood D.E., Salzberg S.L. (2014). Kraken: ultrafast metagenomic sequence classification using exact alignments. Genome Biol..

[41] Sunagawa S. (2013). Metagenomic species profiling using universal phylogenetic marker genes. Nat. Methods.

[42] Segata N. (2012). Metagenomic microbial community profiling using unique clade-specific marker genes. Nat. Methods.

[43] Franzosa E.A. (2018). Species-level functional profiling of metagenomes and metatranscriptomes. Nat. Methods.

[44] Bankevich A. (2012). SPAdes: a new genome assembly algorithm and its applications to single-cell sequencing. J. Comput. Biol..

[45] Peng Y. (2012). IDBA-UD: a de novo assembler for single-cell and metagenomic sequencing data with highly uneven depth. Bioinformatics.

[46] Uritskiy G.V., DiRuggiero J., Taylor J. (2018). MetaWRAP-a flexible pipeline for genome-resolved metagenomic data analysis. Microbiome.

[47] Zhou Q., Su X., Ning K. (2014). Assessment of quality control approaches for metagenomic data analysis. Sci. Rep..

[48] Zhou Q. (2018). RNA-QC-chain: comprehensive and fast quality control for RNA-Seq data. BMC Genom..

[49] Rognes T. (2016). VSEARCH: a versatile open source tool for metagenomics. Peer J..

[50] Lu, J. and S.L. Salzberg, Ultrafast and accurate 16S microbial community analysis using Kraken 2. bioRxiv, 2020: p. 2020.03.27.012047. 10.1186/s40168-020-00900-2 PMC7455996 32859275

[51] Hillmann B. (2018). Evaluating the Information Content of Shallow Shotgun Metagenomics. Msystems.

[52] Johnson J.S. (2019). Evaluation of 16S rRNA gene sequencing for species and strain-level microbiome analysis. Nat Commun..

[53] Haft D.H. (2018). RefSeq: an update on prokaryotic genome annotation and curation. Nucleic Acids Res.

[54] Integrative, H.M.P.R.N.C., The Integrative Human Microbiome Project. Nature, 2019. 569(7758): p. 641-648. 10.1038/s41586-019-1238-8 PMC6784865 31142853

[55] Thompson L.R. (2017). A communal catalogue reveals Earth's multiscale microbial diversity. Nature.

[56] McDonald D. (2018). American Gut: an Open Platform for Citizen Science Microbiome Research. mSystems.

[57] Kodama Y. (2012). The Sequence Read Archive: explosive growth of sequencing data. Nucl. Acids Res..

[58] Meyer F. (2008). The metagenomics RAST server - a public resource for the automatic phylogenetic and functional analysis of metagenomes. BMC Bioinf..

[59] Harrison P.W. (2019). The European Nucleotide Archive in 2018. Nucl. Acids Res..

[60] Chen I.A. (2019). IMG/M vol 5.0: an integrated data management and comparative analysis system for microbial genomes and microbiomes. Nucl. Acids Res..

[61] Zhang, T., et al., MPD: a pathogen genome and metagenome database. Database (Oxford), 2018. 2018. 10.1093/database/bay055 PMC6007212 29917040

[62] Yilmaz P. (2011). Minimum information about a marker gene sequence (MIMARKS) and minimum information about any (x) sequence (MIxS) specifications. Nat. Biotechnol..

[63] Buttigieg P.L. (2016). The environment ontology in 2016: bridging domains with increased scope, semantic density, and interoperation. J. Biomed. Semantics..

[64] Ten Hoopen P. (2017). The metagenomic data life-cycle: standards and best practices. GigaScience.

[65] Wu S. (2020). GMrepo: a database of curated and consistently annotated human gut metagenomes. Nucl. Acids Res..

[66] Shi W. (2019). gcMeta: a Global Catalogue of Metagenomics platform to support the archiving, standardization and analysis of microbiome data. Nucl. Acids Res..

[67] Gonzalez A. (2018). Qiita: rapid, web-enabled microbiome meta-analysis. Nat. Methods.

[68] McDonald D. (2019). redbiom: a Rapid Sample Discovery and Feature Characterization System. mSystems.

[69] Su X. (2018). Identifying and Predicting Novelty in Microbiome Studies. MBio.

[70] Jing G. (2019). Dynamic Meta-Storms enables comprehensive taxonomic and phylogenetic comparison of shotgun metagenomes at the species level. Bioinformatics.

[71] Su X. (2014). GPU-Meta-Storms: computing the structure similarities among massive amount of microbial community samples using GPU. Bioinformatics.

[72] Costea P.I. (2017). Towards standards for human fecal sample processing in metagenomic studies. Nat. Biotechnol..

[73] Hacquard S. (2015). Microbiota and Host Nutrition across Plant and Animal Kingdoms. Cell Host Microbe.

[74] Lozupone C.A. (2013). Meta-analyses of studies of the human microbiota. Genome Res..

[75] Voigt A.Y. (2015). Temporal and technical variability of human gut metagenomes. Genome Biol..

[76] Statnikov A. (2013). A comprehensive evaluation of multicategory classification methods for microbiomic data. Microbiome.

[77] Gevers D. (2014). The treatment-naive microbiome in new-onset Crohn's disease. Cell Host Microbe.

[78] Teng F. (2015). Prediction of Early Childhood Caries via Spatial-Temporal Variations of Oral Microbiota. Cell Host Microbe.

[79] Sun Z. (2019). A Microbiome-Based Index for Assessing Skin Health and Treatment Effects for Atopic Dermatitis in Children. mSystems.

[80] Huang S. (2014). Predictive modeling of gingivitis severity and susceptibility via oral microbiota. ISME J..

[81] Duvallet C. (2017). Meta-analysis of gut microbiome studies identifies disease-specific and shared responses. Nat. Commun..

[82] Jackson M.A. (2018). Gut microbiota associations with common diseases and prescription medications in a population-based cohort. Nat. Commun..

[83] Su X. (2020). Multiple-Disease Detection and Classification across Cohorts via Microbiome Search. mSystems.

[84] Zitnik M. (2019). Machine Learning for Integrating Data in Biology and Medicine: Principles, Practice, and Opportunities. Inf. Fusion.

[85] Fiannaca, A., et al., Deep learning models for bacteria taxonomic classification of metagenomic data. BMC Bioinformatics, 2018. 19(Suppl 7): p. 198. 10.1186/s12859-018-2182-6 PMC6069770 30066629

[86] Kather J.N., Calderaro J. (2020). Development of AI-based pathology biomarkers in gastrointestinal and liver cancer. Nat. Rev. Gastroenterol. Hepatol..

[87] LaPierre N. (2019). MetaPheno: A critical evaluation of deep learning and machine learning in metagenome-based disease prediction. Methods.

[88] Wei Y. (2016). HCP: A Flexible CNN Framework for Multi-Label Image Classification. IEEE Trans. Pattern Anal. Mach. Intell..

[89] He Y. (2018). Regional variation limits applications of healthy gut microbiome reference ranges and disease models. Nat. Med..

[90] Bikel S. (2015). Combining metagenomics, metatranscriptomics and viromics to explore novel microbial interactions: towards a systems-level understanding of human microbiome. Comput. Struct. Biotechnol. J..

[91] Bashiardes S., Zilberman-Schapira G., Elinav E. (2016). Use of Metatranscriptomics in Microbiome Research. Bioinf. Biol. Insights.

[92] Kleiner M. (2019). Metaproteomics: Much More than Measuring Gene Expression in Microbial Communities. Msystems.

[93] Abubucker S. (2012). Metabolic reconstruction for metagenomic data and its application to the human microbiome. PLoS Comput. Biol..

[94] Garretto A., Hatzopoulos T., Putonti C. (2019). virMine: automated detection of viral sequences from complex metagenomic samples. PeerJ.

[95] McHardy I.H. (2013). Integrative analysis of the microbiome and metabolome of the human intestinal mucosal surface reveals exquisite inter-relationships. Microbiome.

[96] Franzosa E.A. (2014). Relating the metatranscriptome and metagenome of the human gut. Proc. Natl. Acad. Sci. U S A.

[97] Narayanasamy S. (2016). IMP: a pipeline for reproducible reference-independent integrated metagenomic and metatranscriptomic analyses. Genome Biol..

[98] Quinn R.A. (2016). From Sample to Multi-Omics Conclusions in under 48 Hours. mSystems.

[99] Rinke C. (2014). Obtaining genomes from uncultivated environmental microorganisms using FACS-based single-cell genomics. Nat. Protoc..

[100] Ho C.S. (2019). *Rapid identification of pathogenic bacteria using Raman spectroscopy and deep learning.* Nature. Communications.

[101] Teng L. (2016). Label-free, rapid and quantitative phenotyping of stress response in E. coli via ramanome. Sci. Rep..

